# Endoscopic Surgical Excision of Ectopic Tooth in Left Nasal Cavity

**DOI:** 10.7759/cureus.17465

**Published:** 2021-08-26

**Authors:** Soumya Gupta, Michael Cipolla

**Affiliations:** 1 Otolaryngology, Jacobs School of Medicine and Biomedical Sciences, University at Buffalo, Buffalo, USA

**Keywords:** ectopic tooth, intranasal tooth, nasal endoscopic surgery, supernumerary teeth, deciduous teeth, endonasal endoscopic surgery, general otolaryngology

## Abstract

Ectopic teeth in the nasal cavity are a rare phenomenon. They are often associated with a variety of symptoms and future complications, ranging from nasal crusting and obstruction to chronic infections. In most reported cases, their removal is recommended. Here, we report a case of an ectopic intranasal tooth in a symptomatic adult. The tooth was removed endoscopically with good results.

## Introduction

The presence of an intranasal tooth is uncommon. Ectopic intranasal teeth may be permanent, deciduous, or supernumerary [1]. The estimated incidence of any supernumerary teeth in the population is 0.1-1%, with the most common location being the upper central incisor area. An even smaller percentage of these teeth is found within the nasal cavity [2]. There are reports of some ectopic teeth being etiologically linked to trauma [3,4]. Regardless of the etiology, identifying an intranasal ectopic tooth is necessary in order to minimize potential complications. Intranasal teeth can be asymptomatic and discovered incidentally, but patients may also report symptoms including foul-smelling discharge, nasal obstruction, chronic rhinosinusitis, and epistaxis, among other complaints [1-6]. Surgical removal of the tooth may thus benefit the patient by resolving symptoms and reducing the potential for complications. We present a case of an adult with a symptomatic ectopic intranasal tooth and report our technique for endoscopic removal and clinical outcomes.

## Case presentation

A 39-year-old female, with a history of chronic rhinitis, presented in June 2020 after several years of left-sided green nasal crusting and discharge. She denied sinus pain, epistaxis, or difficulty with nasal breathing. The patient ultimately came for evaluation because a family member commented on a bad smell from her nose.

Of historical significance, the patient recalled breaking her nose and knocking out her front teeth in a roller-skating accident as a young child. The patient could not recall whether this dental trauma affected permanent teeth or deciduous teeth, and she did not remember which front teeth were involved. No history of maxillofacial surgical intervention or further trauma was reported, and past medical history was otherwise noncontributory.

**Figure 1:**
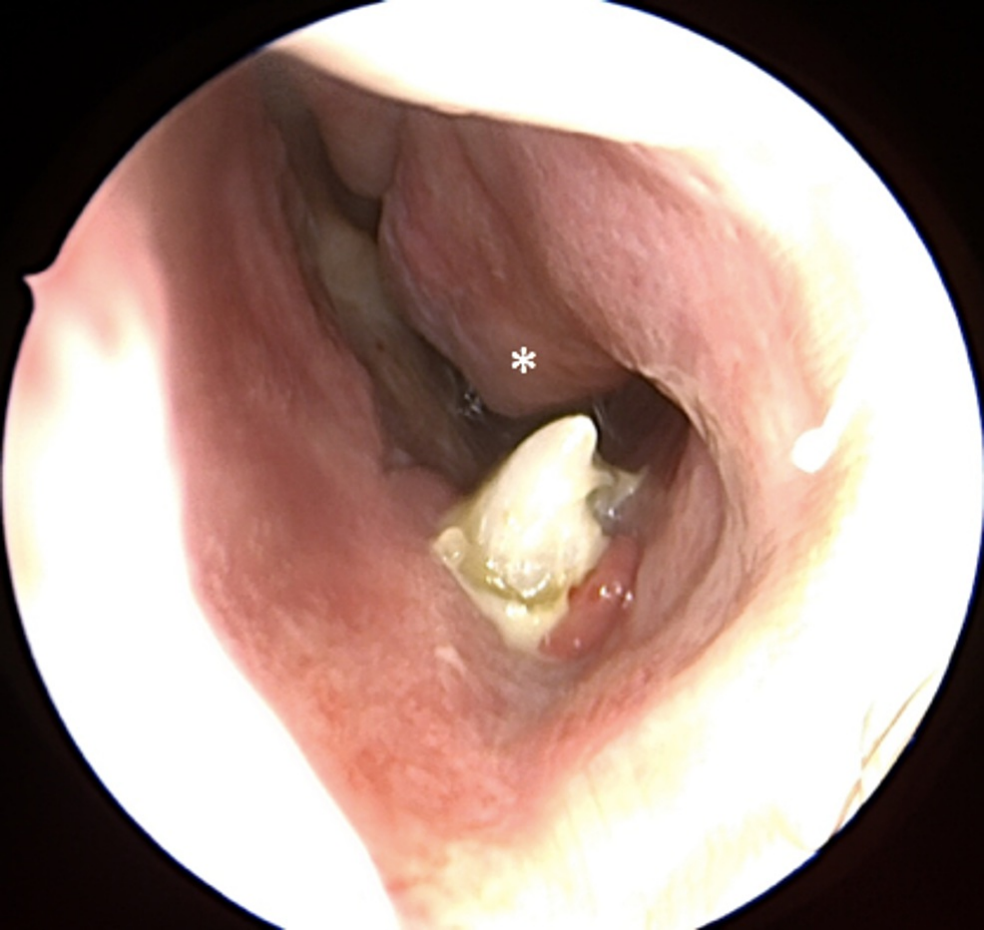
Nasal endoscopy reveals a tooth root extending from the floor of the left nasal cavity and projecting toward the inferior turbinate (*). Crusting is present.

**Figure 2:**
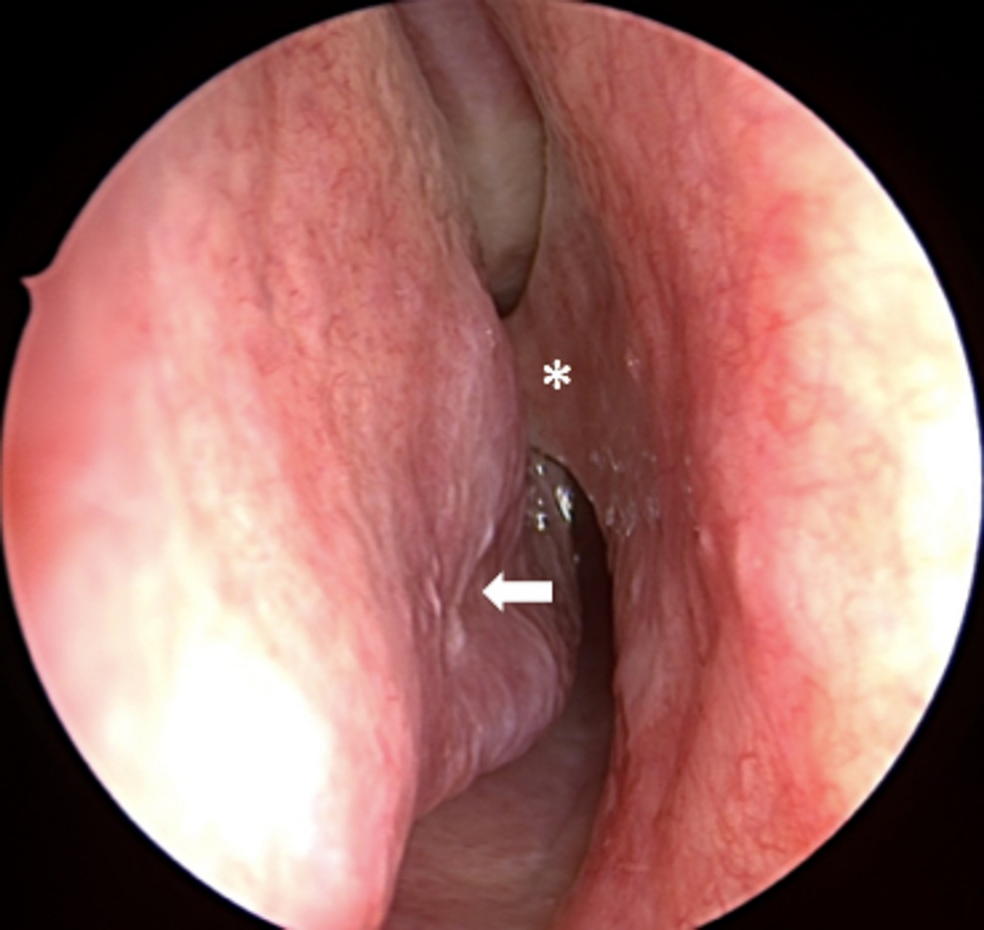
In the right nasal cavity, nasal septal deviation to the right is seen, with a bony spur (*) abutting the right inferior turbinate (white arrow).

Nasal endoscopy revealed a pearly white, hard mass extending from the floor of the left nasal cavity. Gentle palpation of the mass demonstrated mobility. The tip was seen to abut against the left inferior turbinate (Figure 1). Additional endoscopic findings included inferior turbinate hypertrophy, and nasal septal deviation to the right, with a spur projecting to the right (Figure 2). The remainder of the head and neck examination was unremarkable, with permanent dentition, including all four maxillary incisors, visible.

**Figure 3:**
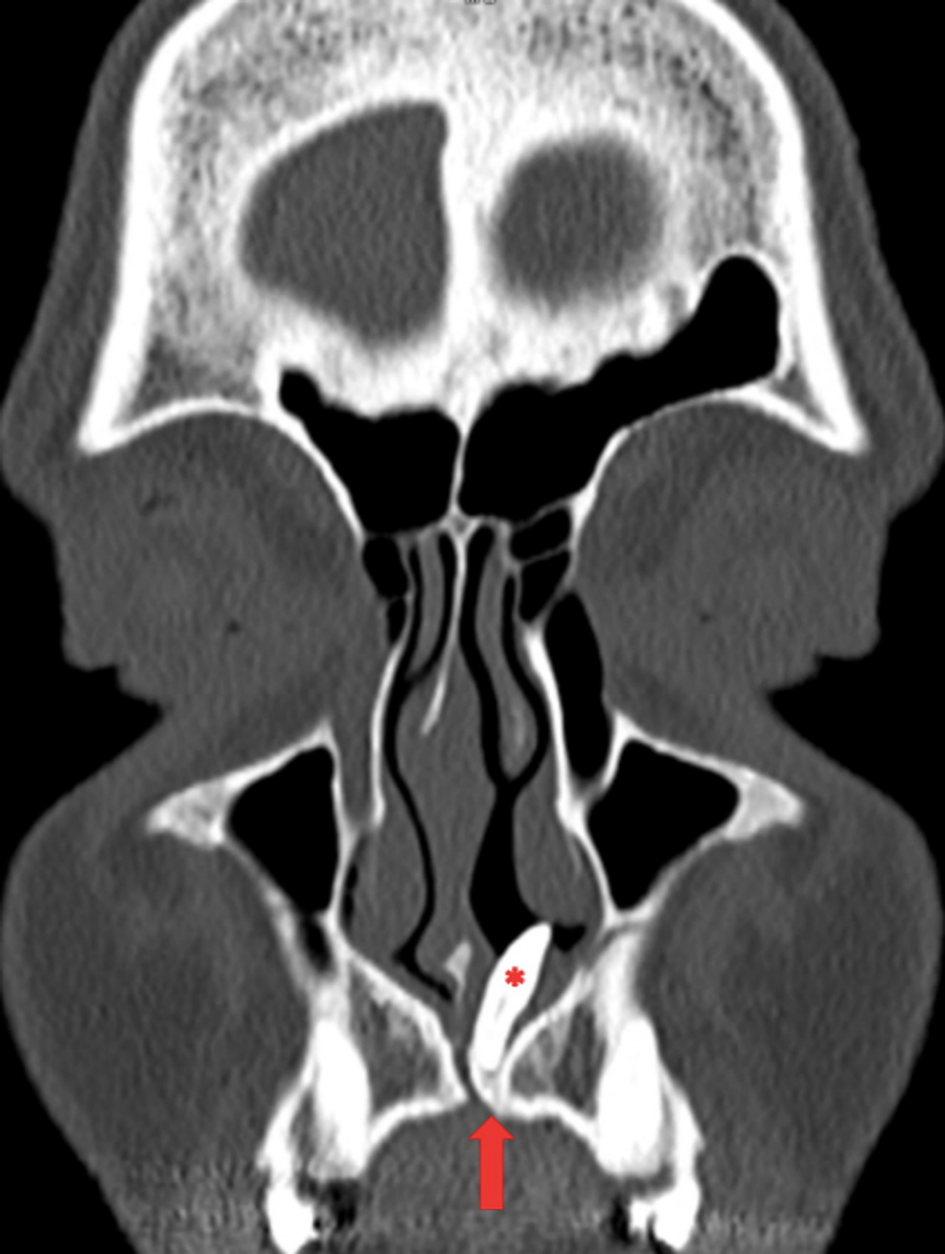
Coronal CT of sinuses confirms the presence of an ectopic nasal tooth (*), extending from the incisive canal (red arrow) into the nasal cavity.

Computed tomography (CT) imaging of the sinuses confirmed the presence of an ectopic tooth, with a poorly-developed crown in the incisive canal and the root extending into the nasal cavity (Figure 3). The tooth did not appear to have any bony attachment, contributing to its mobility. No associated cystic components or soft tissue masses were observed.

**Figure 4:**
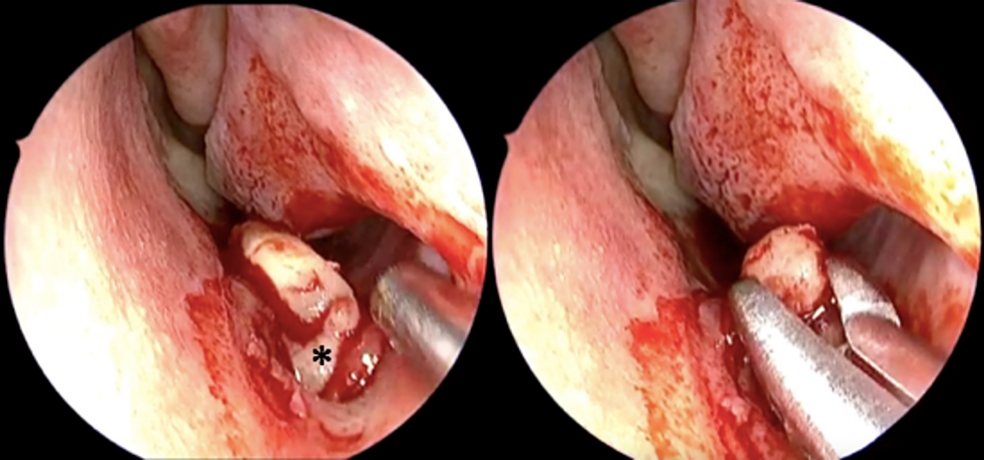
Forceps extract the tooth from its attachment in the nasal mucosa (*).

The patient underwent endoscopic removal of the intranasal tooth, septoplasty, and inferior turbinate reduction under general anesthesia. The tooth was gently loosened from its soft tissue attachment and removed with a Blakesley forceps (Figure 4). Curettage of the extraction site was performed.

**Figure 5:**
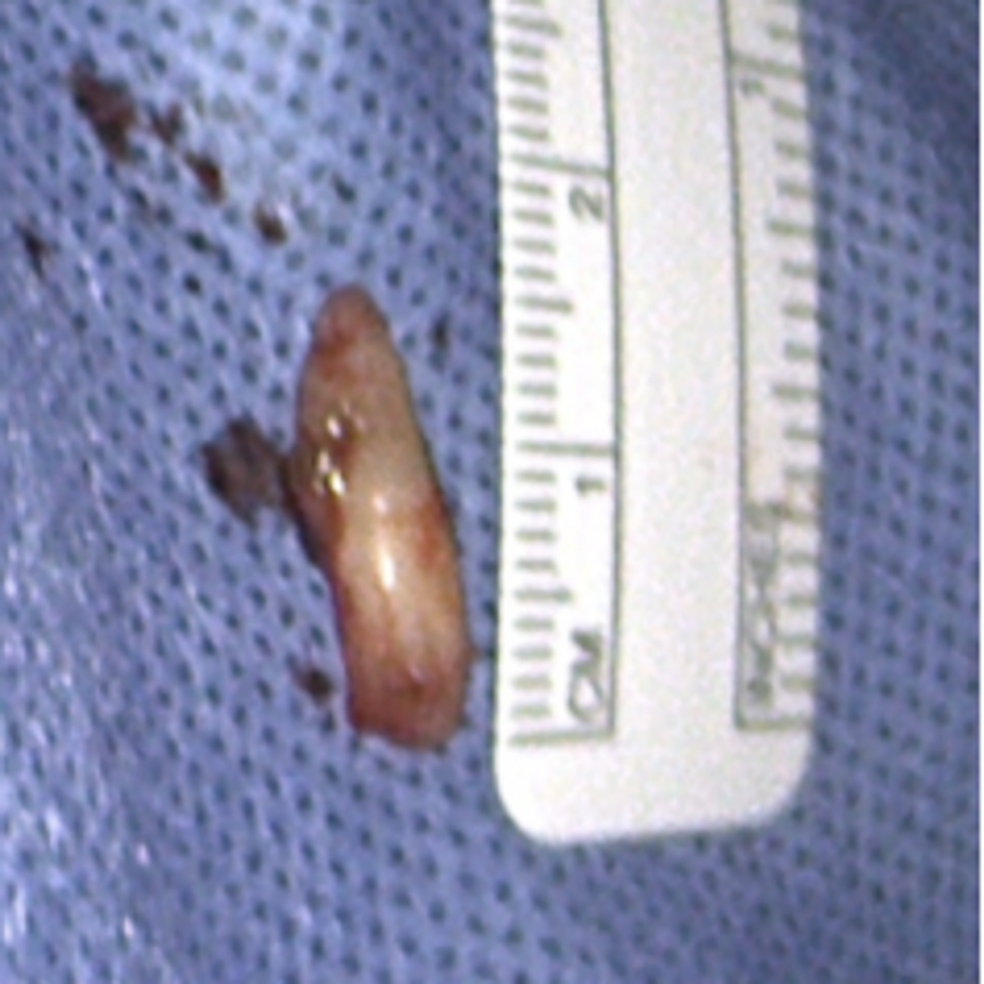
Measurement of the extracted intranasal tooth.

The removed tooth was approximately 1.6 cm in length and incompletely developed (Figure 5). All surrounding soft tissue removed was benign, and the nasal cavity floor was left intact.

Postoperatively, the nasal mucosa healed well, with no further crusting, and resolution of symptoms. The patient had no postoperative dental paresthesia.

## Discussion

The cause of ectopic tooth eruption and growth is not well understood. Reports of some ectopic teeth have been etiologically linked to displacement by trauma or cysts, while others are associated with congenital conditions, including cleft palate and Gardner syndrome [3,4]. Although we have an incomplete timeline of this patient’s maxillofacial childhood injury, her ectopic tooth is most likely supernumerary in nature. This is supported by the findings of tooth mobility without bony attachment, the presence of both central and lateral maxillary incisors present on physical examination, and the incomplete development of the removed tooth. Displacement of the tooth could have been traumatic, given her previous history. However, displacement of a deciduous incisor into the nasal cavity cannot be fully ruled out.

Regardless of the etiology and despite its rare prevalence, identifying an intranasal ectopic tooth is necessary in order to resolve symptoms and minimize potential complications. Although sometimes asymptomatic, an intranasal tooth left alone could potentially serve as a nidus for infection, mineralization, and debris [1], given its relatively exposed location in the nasal cavity and potentially years-long presence. As in this patient’s case, the tooth’s presence likely resulted in nasal malodor, crusting, and discharge. Other patients have been reported to suffer from chronic nasal obstruction and congestion, facial pain, purulent or blood-tinged rhinorrhea, recurrent epistaxis, rhinitis caseosa, and fungal ball development [1-7].

Surgical removal, guided by either endoscope or headlights and nasal speculum, is a safe and effective intervention [1,3,4,6,8]. In one systematic review of 23 studies, endoscopic sinonasal surgery was used to remove ectopic teeth from either the nasal cavity or maxillary sinus. As with our patient, all those who initially presented with symptoms had complete resolution after surgical removal, with no reported post-operative or long-term complications [9]. While removal under both direct headlight visualization and endoscope have shown good outcomes, the endoscopic approach provides the added benefit of good illumination, clear visualization, and more precise dissection [1]. This procedure is beneficial in resolving symptoms and preventing complications, with minimal operative risk to the patient.

## Conclusions

An ectopic intranasal tooth is a rare phenomenon. While its etiology is not well understood, it is often associated with trauma or congenital conditions. Regardless of the cause, the presence of an intranasal tooth may result in a variety of symptoms and potential complications. An endoscopic removal is a viable option that may eliminate further risks, with minimal harm to the patient.
